# Assessing the health risks of consuming ‘sachet’ alcohol in Acoli, Uganda

**DOI:** 10.1371/journal.pone.0212938

**Published:** 2019-02-27

**Authors:** Ochan Otim, Tom Juma, Olara Otunnu

**Affiliations:** 1 Department of Humanities and Sciences, University of California—Los Angeles, Los Angeles, California, United States of America; 2 Environmental Monitoring Division, City of Los Angeles, Playa Del Rey, California, United States of America; 3 Former Under-Secretary-General of the United Nations, New York, New York, United States of America; Northumbria University, UNITED KINGDOM

## Abstract

The increased mortality rate among the Acoli people of northern Uganda is anecdotally blamed on excessive consumption of cheap and widely available sachet-packaged alcohol in the region. In this paper, we quantify this perceived association by determining statistically the health risks associated with ingesting 20 heavy metals in 17 popular spirits products consumed in Acoli. Thirteen of these products were industrially packaged in sachets (locally known as ‘sachet,’ *waragi*, *arege* or *moo lyec*) and four were locally produced *Lira-Lira* spirits from Bolo, Awere and Teso Bar in the region and Nsambya in southern Uganda. A Scottish whisky purchased in San Diego (USA) was our reference. Risk assessment was performed according to standardized protocols developed by the United States Environmental Protection Agency (US EPA). Our results show that a strong correlation indeed exists between health risks and ingestion of spirits in Acoli. At >2.5 sachets/day for 240 day/year over a lifetime for example, the risk of developing *cancer* due to exposure to As, Pb and Cr alone is 1 in 102,041. This estimate excludes ethanol, a known carcinogen, and 17 heavy metals also studied due to lack of their cancer slope factors. The primary *non-cancer* related health risk factor in all samples tested is ethanol with unacceptably high health index of four. The *Lira-Lira* spirits, with 100–6000% copper above the US EPA limit for intake by oral ingestion in water, would be the ‘cleanest’ without copper and at par with the Scottish whisky. Collectively, we find that no amount of alcohol consumed in Acoli is safe. Preventive measures are therefore recommended to reduce mortality in Acoli in particular, and in Uganda in general. These measures should include public education, better public policies, creating productive economic activities other than brewing alcohol, and social activities that engage people away from drinking.

## Introduction

The sub-Saharan countries of Africa are currently experiencing an increase in adult mortality partly believed to be due to the heavy consumption of cheaply and widely available spirits in the region [1,2]. This scenario is best illustrated by the plight of the Acoli population of Uganda [3], a community in which uncontrolled alcohol consumption and alcohol dependency was acquired involuntarily during a 20-year civil war. This population, centered approximately 350 km (220 mi) north of Kampala, the capital city (S1 Fig), is emerging from two decades of interment in which the entire population (close to 2 million people) were confined into heavily guarded satellite camps [3–8]. Within these camps, distilled or refined alcohol dependency was allowed to take root, albeit disproportionally affecting twice as many adult males as women [8]. Since leaving the internment camps, this population continues to experience high death rates which the local population blamed on the consumption of the poorly regulated and readily available spirits in industrially packaged plastic sachets or tot packs of 100 mL or less [3,8–10]. The suspected linkage between mortality and alcohol consumption is strengthened by an incident in 2008 where over 100 deaths were officially attributed to alcohol poisoning in one locality alone [11]. For this reason and others, the Acoli population of Uganda are not only right in suspecting spirits, but their predicament also offers uniquely an opportunity to study a host of health-related factors associated with physical confinement and heavy alcohol consumption.

Historically, the cultural or recreational consumption of alcoholic beverages (wholly unrefined or ‘opaque beers’ in current lexicon) in Acoli was a highly controlled social affair, going as far back as the ‘discovery’ of fermentation (immortalized in a classic Acoli play *Yom Cwiny Oneko Latina*–Generosity Kills the Generous One [12]). Most of these opaque beers (*lacoi/malwa* [13] and *kwete* [14] in Uganda, and several in Tanzania [15]) are not only rich in nutrients [16,17], they have no known side effects. Some believe that they may actually be less harmful because of their low alcohol contents [15] and bulkiness (not pure) [16,17] which prevents overconsumption of alcohol thereby minimizing health risk. With technological advancements however, these opaque beers have been largely replaced by industrially distilled and blended spirits sold in easily affordable sachets or tots [3]. Ethyl alcohol (ethanol), the main intoxicant in the sachets, has no known nutritional values, but has strong physiological effects. The sachets/tot packaged spirits do not only have higher concentrations of ethanol than the opaque beers (up to 40% by volume), but contain toxic metals (as shown here) and organic pollutants as well (*unpublished* material), or blended with toxic substances by unscrupulous producers and sellers of alcohol. The toxic metals and organic pollutants in most cases are carry-overs from the production processes although some are acquired through exposure to polluted environment. Compounding the problem of alcohol dependency is the fact that the local Acoli population has also perfected the art of distilling alcohol from the opaque beers, the products of which are now widely available alongside the industrially packaged sachet spirits [personal experience]. These undocumented distillates are locally known as *waragi*, a name derived from *war gin*, the original British settlers’ name. *Waragi* is also variously referred to as *moo lyec* or *arege* in Acoli, *Lira-Lira* in Lango and *Kasese-Kasese* in western Uganda.

### Alcohol regulation in Uganda

Lacking in the technological advancement leading to the distillation of opaque beers is, unfortunately, a parallel development of regulations to address the explosion of the cheap supply of refined alcohol and the attendant alcohol dependency that comes with it [18]. (Regrettably, Uganda is not alone here in Africa [19,20].) At the writing of this paper, a willingness to ban the packaging of *waragi* in sachet in an attempt to limit the ease with which alcohol can be accessed and distributed has been declared by the Uganda Ministry of Trade, Industry and Cooperatives [21,22]. The declaration identifies ‘health and environmental problems caused by the abuse of alcohol production’ as the reason for banning sachet alcohol. While this is a good first step towards protecting vulnerable populations, a direct link between the quality of the drinks and health risks would have added weight to the declaration. After all, alcohol regulation policies in Uganda have an association with alcohol dependency [23]. We believe this omission, just like in the rest of sub-Sahara Africa, is due to the absence of scientific data to backup pronouncements. The current Uganda Standard (adopted verbatim from the East African Standard) is awfully inadequate in this context because it regulates only the lead content of *waragi* [24]. Yet (as will be shown here) lead is not a major health risk in Uganda’s *waragi* in comparison to other heavy metals also found in *waragi*, or ethanol (a heavy metal is defined as any toxic metal regardless of its atomic mass [25]). Interestingly, food contamination by heavy metals is recognized as a serious health risk in Uganda [26] but not alcoholic drinks to the same extent.

### Ethanol, a prime health risk suspect

The causes of increasing mortality in Acoli could be attributed to many factors. However, since alcohol is now a bona fide carcinogen [27], and the causal relationships between alcohol consumption and cancer of the larynx [28,29], esophagus [28–30], liver [28,29,31,32] are well established, it is only appropriate that the role of ethanol itself is emphasize in studies such as this one. Indeed, no amount of alcohol appears to be safe. For example, 3.4×10^4^ cancer related deaths were attributed to light drinking in a study published in 2013 [33]. On the other hand, alcohol consumption is also associated with a decrease in cancer of the kidney and non-Hodgkin lymphoma [34,35]. These benefits, however, are outweighed by harms done.

### Study design

In this paper, our main goal is to initiate an investigation into the link between alcohol consumption and health risk in Uganda in general and Acholi region in particular. Our approach is to assess the health risk associated with consuming heavy metal contaminated alcoholic products sold to the former internment camp residences. The heavy metals studied are silver (Ag), arsenic (As), aluminum (Al), barium (Ba), beryllium (Be), cadmium (Cd), cobalt (Co), chromium (Cr), copper (Cu), manganese (Mn), molybdenum (Mo), nickel (Ni), lead (Pb), antimony (Sb), selenium (Se), tin (Sn), vanadium (V), thallium (Tl), strontium (Sr) and zinc (Zn). As, Pb, Hg, and Cd are 1^st^, 2^nd^, 3^rd^ and 7^th^ on the United State Agency for Toxic Substances and Disease Registry priority list of substances which pose the most significant potential threat to human health [36]. We hope that our findings will help authorities in Uganda in developing the necessary and appropriate alcohol quality assurance standards and enforcement policies to minimize health risks associated with unsafe alcohol consumption.

## Materials and methods

### Sampling and quality control

Thirteen (13) brands of 100-mL sachet-packaged spirits used for this study (Table 1) were purchased from the study area (S1 Fig). These purchases were done without attempts to pick true random samples because of the logistical challenges and the restrictions encountered in attempts to collect representative samples of the sachet types sold and consumed in the study area. That said, as much diversity as possible was included on a first encounter basis. The 13 brands were Big 5 Vodka (B5V), Beckham Spirit (BEG), Bond 7 Whisky (B7W), Brigade Spirit (BRG), Chief Waragi Spirit (CW1 and CW2, duplicates sampled one year apart), Goal Vodka (GOV), Kick Spirit Pineapple Waragi (KPW), Relax (REX), Royal Vodka (ROV), Salongo Spirit (SAG), Uganda Waragi (UGW), and V6 Tangawizi Vodka (V6T). At the time of this study, spirits sold in large bottles were outside the financial reach of the study population, and hence of no consequence here. For comparison, *waragi* from undocumented distillation processes (here referred to interchangeably as *Lira-Lira* spirits) were collected in duplicates in new and unused 50-mL Falcon screw-capped polypropylene conical centrifuge tubes (Fisher Scientific, Waltham, MA, USA) from three study area Bolo (BOL), Awere (AWE), and Teso Bar in Lira (TEB), and from a Kampala suburb (NSB, Nsambya Police Barack; S1 Fig). To ensure that the Falcon tubes were not introducing extractable metals into samples, two fresh tubes were filled with a 50/50 (v/v) mixture of 100% ethanol/distilled water and incubated for three months, the contents of which were analyzed as regular samples. A well characterized Scotch whisky (The Glenlivet (TGL) [37]) purchased in San Diego, California (USA) was used as a ‘certified reference material.’ To ensure control over the reference conditions under which trace amounts of metals could be detected with 99% confidence in ethanol, the 50/50 (v/v) mixture of 100% ethanol/distilled water was included as a blank sample in each batched analysis, and as a platform for laboratory control spiked duplicate samples.

**Table 1:** Description of the alcohol samples used in this study.

ID	Name on sachet	Blended and Packaged by	Composition as written on sachet
UGW	Uganda Waragi	EABL Group at Uganda Breweries.	40%ABV.
B7W	BOND 7 Whisky	EABL Group at Uganda Breweries.	40%ABV.
CW1	CHIEF WARAGI (GIN)	Plot No. 872 & 873, KLA, UG.	40% Vol., Extra neutral alcohol, purified water and permitted food flavours.
CW2	CHIEF WARAGI (GIN)	Plot No. 872 & 873, KLA, UG.	40% Vol. Purified water, Extra neutral alcohol, and permitted food flavours.
SAG	SALONGO GIN	Parambot Distilleries Ltd., KLA, UG.	40%ABV Neutral Spirit, Water & Flavour.
ROV	Royal Vodka	Parambot Distilleries Ltd., KLA, UG.	40% Vol. Neutral Spirit, Water & Flavour.
KPW	KICK Gin Pineapple Waragi	Brigade Distilleries Limited, KLA, UG.	40% Alc./Vol., Portable spirit, purified water and permitted food flavours.
BRG	BRIGADE GIN	Brigade Distilleries Limited, KLA, UG.	40% Alc./Vol., Portable spirit, purified water and permitted food flavours.
B5V	BIG 5 VODKA	King Albert Distilleries Ltd, KLA, UG.	40%V/V. Water, Potable Alcohol, Permitted Food Flavour.
REX	Relax	King Albert Distilleries Ltd, KLA, UG	20% Alc./Vol. Extra Neutral Spirit, Purified Water, Permitted Food Flavour and Cream.
GOV	Goal Vodka	HEMA BEVERAGES LTD Plot No. 923, KLA, UG.	40% Vol., Portable Spirit, purified water and permitted food flavours.
BEG	BECKAM Gin	Boss Beverage International (BBI) Ltd, KLA, UG	40% Vol. ALC. Neutral spirit, Gin Flavour, Purified Water.
V6T	V6 Tangawizi vodka	Blue Nile Distilleries Ltd, KLA, UG.	40% Vol. Purified water, portable spirit, and permitted food flavours.
AWE	Awere*	Lira-Lira, Awere Trading Center, AWERE, UG.	Unknown.
BOL	Bolo*	Lira-Lira, Bolo Trading Center, BOLO, UG.	Unknown.
TEB	Teso Bar*	Lira-Lira, Teso Bar, LIRA, UG.	Unknown.
NSB	Nsambya*	Lira-Lira, Nsambya Police Barrack, KLA, UG.	Unknown.
TGL	The GLENLIVET **	George & J.G. Smith, Scotland [37].	75cl 750ml 40%vol 40^o^GL 40%alc./vol.

* Indigenously brewed and distilled ethanol, locally known as Lira-Lira (sampled in duplicates in capped 50mL Corning media tubes).

** Single Malt Scotch Whisky purchased in San Diego, California USA. KLA, UG: Kampala, Uganda.

### Sample preparation

Each sachet alcohol sample and the TGL reference were transferred into a pair of 50-mL Falcon centrifuge tubes to maintain consistency in sample handling with the *Lira-Lira* spirits. Twenty (20) mL of each sample was mixed with 0.4 mL of 50% nitric acid in distilled H_2_O and 0.2 mL of 50% nitric acid in distilled H_2_O hydrochloric acid (Thermo Fisher Scientific, Waltham, MA, USA) in a digestion tube and digested on a heating block (Environmental Express, Charleston, SC, USA) by refluxing at 93°C for 2 h. The digested samples were allowed to cool down to room temperature, filtered (No. 40 Whatman filter paper, Thermo Fisher Scientific, Waltham, MA, USA), and adjusted back to 20 mL with distilled/deionized water.

### Health risk assessment

In calculating risks, our working assumption is that a contaminated alcoholic drink exposes a consumer to a mixture of metals which could potentially interact and produce synergistic effects in humans (in blood for example, non-essential elements such as Tl, Ni, and Al are strongly competitive with elements required for the proper functions of the body such as Ca, Mg, Zn, Se, Mn, Co, Cr, and Mo [38]). The overall picture of metals contamination in each *waragi* brand is, therefore, important to understanding the potential health risks associated with a *waragi* brand. The United States Environmental Protection Agency (US EPA) proposed a method for estimating such exposure risk in 1986 (with subsequent Supplemental additions in 2000) and developed the target hazard quotient *(*the *THQ* system) for this purpose [39,40]. *THQ* is the ratio of the level of a chemical pollutant in a matrix of interest to a US EPA predetermined *oral reference dose* of that chemical, taking into account the length and the frequency of exposure, the body weight of the consumer and the amount ingested. We used the *THQ* system to estimate the risk of consuming alcohol in northern Uganda. Strange as it may sound, ethanol, the major intoxicant in *waragi*, is rarely included in estimating risks associated with consuming alcoholic drinks despite ample evidence pointing to ethanol as a carcinogen [27,29,41]. As mentioned earlier, we are not following that example here.

The US EPA procedural guideline for evaluating health risks data associated with exposures to chemical mixtures recommends following these four steps in sequence [39,40]: hazard identification, the daily hazard intake estimation, risk estimation and risk characterization. We adopted this approach to make alcohol consumption dose-response assessment and risk characterization as described in the following four subsections.

#### Chemical analysis

The first step in health risk assessments is hazard identification. This is principally a qualitative and a quantitative chemical analysis step aimed at identifying any relevant chemical species in a target matrix and quantifying their extent of contamination. In this study, twenty metals listed in the introduction section were investigated as possible hazards for alcohol consumers in northern Uganda as follows. The 20-mL digest obtained after sample preparation were analyzed for the metals using a Thermo Scientific X-Series 2 Inductively Coupled Plasma-Mass Spectroscopy instrument (ICP-MS, Thermo Electron North America LLC) set to run on both the standard mode, and the Collision Cell Technology (CCT) and Kinetic Energy Discrimination (KED) mode [42]. The CCT-KED mode greatly reduces interferences, especially those due to isobaric polyatomic ions. The performance of instrument was optimized with a solution containing 10 μg/L each of Li, Be, Co, In, Ba, Ce, Pb, Bi, Tl, Mg, Rh, Y, and U. For quantitation, a 5-point calibration curve (5, 10, 20, 100 and 200 μg/L levels) was generated for each metal. In addition, a 0.20 μg/L lowest point was added for Cd and Ag (levels of Ag is 1/8^th^ of all the other elements because of its low solubility), a 0.5 μg/L lowest point for Sb, Be, Cr, Cu, Pb and 1 μg/L lowest point for As, Se, Tl, Ni and Zn. The accuracy of measurements was ascertained by analyzing standards from a second source in the same batch. This was verified dynamically after every 10 samples to ensure against instrumental drift or artefactual contamination. Precision was assured by analyzing samples in duplicates. All primary metal standards were certified references obtained from SPEX Certiprep (Metuchen, NJ, USA) and Inorganic Ventures (Christiansburg, VA, USA). The detection limits are given in S1 Table. In data analysis, interquartile range (IQR) was used to indicate where most of the measured levels lie. IQR was calculated by subtracting the 25^th^ percentile (Q_1_) from the 75^th^ percentile (Q_3_). Half of a sample readings fall between Q_1_ and Q_3_; a quarter of the readings are smaller than Q_1_, and a quarter higher than the Q_3_.

#### Estimation of the average daily heavy metal intake

Exposure assessment to estimate the intensity, frequency, and duration of human exposures to alcohol contaminated with metals was carried out by calculating the average daily intake (*ADI*, in mg/kg/day) of the 20 metals studied, and of ethanol ingested by an Acoli adult (assuming consumption in this population begins at age 15) using the following equation [39,40]:

$$ADI=\frac{M_{C}\times IR\times 10^{-3}\times EF\times ED}{BW\times ATn}$$

where *M*_*C*_ is the individual concentrations in *mg* of metal per *kg* of original drink; *IR* is 78.54 g/day, the ingestion rate determined by assuming that an adult in the study area consumes 2.5 times a 100-mL of 40% ethanol (v/v) sachet per day. For comparison, one standard alcoholic drink by the World Health Organization is a 30-mL volume of alcoholic drink [43]. The 10^−3^ is a conversion factor. *EF*, the exposure frequency, is 240 day/year on a conservative basis of 5 days of ingestion per week. *ED*, the exposure duration, is 15,932 days based on a life expectancy of 58.65 years less 15 years, the age at which alcohol ingestion in Acoli is assumed to begin (i.e. 365 days/year × [58.65 years—15 years]; see also [19]). *BW*, an adult body weight in Acoli is assumed on reasonable grounds to be 60 kg (Kitgum Hospital, personal communication). *ATn* is the life expectancy of 21,407 days (i.e. 58.65-year lifespan). The list of all parameters used to calculate the health risk is presented in Table 2.

**Table 2:** Exposure parameters used for assessing the health risk of consuming 40% (v/v) sachet ethanol in Acoli, Uganda.

Parameter	Unit	Adult
Ethanol concentration (*M*_*c*_)^a^	mg/kg	4000
Ingestion Rate (*IR*)	mg/day	78540^b^
Exposure Frequency (*EF*)^c^	day/year	240
Exposure Duration (*ED*)	year	43.65
Body Weight (*BW*)	kg	60
Average Exposure Time (*ATn*)^d^	day	21407
Conversion Factor (*CF*)	kg/mg	10^−6^
Average Daily Intake (*ADI*)	mg/kg·day	256
Oral Reference Dose (*RfD*)	mg/kg·day	62
Target Hazard Quotient (*THQ*)	-	4

^a^ Assuming 40% ethanol (v/v) = 40 g ethanol/100 g sample.

^b^ Assuming an adult consumes 100 mL sachet of aqueous 40% ethanol (density of 100% ethanol: 0.7854 g/mL) 2.5 times in a day.

^c^ Assuming an adult consumption rate of 5 days/week.

^d^ Assuming alcohol consumption began conservatively at age 15 and a lifespan of 58 years.

#### Estimation of health risk

The third step in health risk assessment, the dose-response assessment, estimates toxicity resulting from exposure to toxins. The two key toxicity indices we used in this study are the reference dose, *RfD*, and the cancer slope factor, *CSF*. *RfD* is a non-carcinogenic threshold derived from animal studies [44] and is an estimation of the maximum acceptable oral dose of a toxic substance on a human population through daily exposure, taking into consideration a sensitive group during a lifetime [45]. *RfD* for Pb is not established as yet and in this study, a value of 1.5 is used [46]. The ratio of *ADI* to *RfD* is referred to as *THQ*, the target hazard quotient. *THQ* is a decision criterion for non-carcinogenic risk and is often set to 1 (a threshold below which no adverse effects are expected to be observed).

The cancer slope factor, *CSF* (ideally, a potency factor) is calculated based on the knowledge or the probability that a chemical is carcinogenic; it gives the proportional increase in the risk of getting cancer when a dose of a toxin is ingested every day over a lifetime. So far, only three metals (As, Cr(VI) and Pb) have *CSF* values (1.5, 0.5 and 0.0085 mg/kg·day, respectively). *CSF* values, it must be noted, are irrelevant for chemicals that cause the destruction of cellular genetic materials (DNA and RNA) because there are no ‘safe’ levels for carcinogens. Metals such as Sb, Be, Cd, Co, Ni and V together with known carcinogens As, Cr and Pb fall into this category [47,48].

#### Risk characterization

The last step in the health risk assessment is the risk characterization. This step integrates all information from steps one to three to arrive at a quantitative estimate of potential cancerous or non-cancerous health risk [44]. The non-cancerous risk estimate is based on calculated health index (*HI*) obtained in this study by summing up all *THQ* values for individual metals (and ethanol). In summing up *THQ* values, we assume that multiple contaminants results in additive effects. *HI* is interpreted as follows: consumers are safe when *HI* < 1, but are at a level of concern when 1< *HI* < 5 [49].

#### Non-carcinogenic health risk brand similarities

Hierarchical agglomerative cluster analysis (CA) was used to determine inter-brand risk similarities using an 18×19 *THQ* data matrix uploaded into PAST software (PAleontological STatistics; [50]). Copper, found to be an outlier, was excluded. The stability of the resultant clusters was tested by randomly dividing the brands or metals *THQ* values into two halves before repeating CA on each half to see if a similar cluster structures can be found in each half of the samples. The randomizations of the brands or metals *THQ* values were performed using MS Excel 2010 software as follows. Random numbers between 1 and 5000 were assigned to each brand or metal, and then the numbers were sorted from smallest to the largest before dividing the orders into two halves.

## Results

### Quality control

To assure that the secondary containers used to store samples were not themselves contributing metals into samples, two 50-mL Falcon tubes were filled with 50/50 (v/v) 100% ethanol/distilled water mixture and incubated for three months. Results show that the tubes had no detectable levels of metals of interest. This means the sample collection tubes and the manner in which samples were transferred into them from sealed sachets did not introduce extractable metals into the samples. Results also show that the laboratory blank sample (the 50/50 (v/v) 100% ethanol/distilled water solutions) and the laboratory control sample spike duplicates (two fractions of blank solution spiked with the 20 metals of interest), both included in each sample batch, had no unexpected results. This means the reference conditions under which trace amounts of metals could be detected with a 99% confidence in the drinks were under control.

### Descriptive analysis

**Fig 1:**
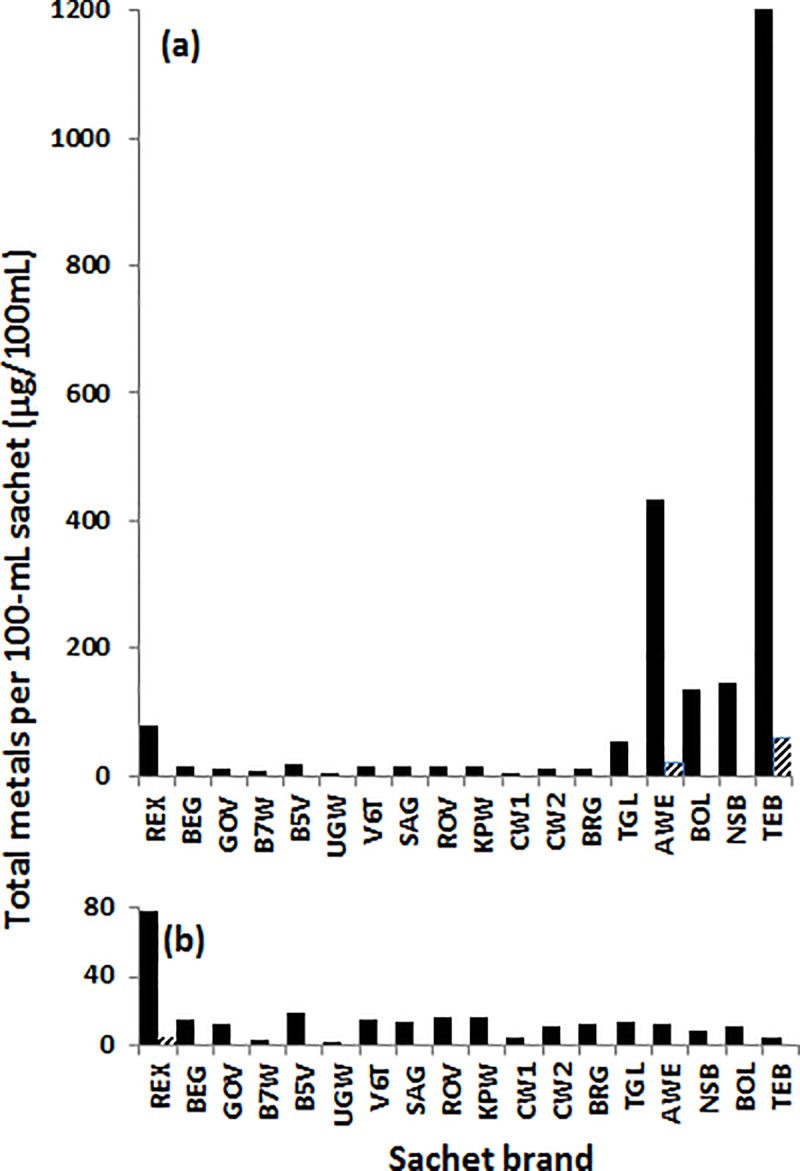
Sum of all metals detected in each sachet is shown as solid rectangles. (a) Cu is detected at high levels in locally distilled alcoholic drinks (AWE, 421 μg/100 mL (97%); BOL, 123 μg/100 mL (92%); NSB, 140 μg/100 mL (94%); TEB, 1210 μg/100 mL (100%) and in the Single Malt Scotch Whisky (TGL, 41 μg/100 mL (76%)); the former due to long copper tubes employed in the distillation process, and the latter is due to copper pots used in the brewing and distillation processes. (b) An expansion of the plot in (a) without Cu, an outlier, to confirm the disproportionate level of copper in the *Lira-Lira* spirits. Note that levels of all metals are overrepresented in REX. Means are very low and shown as angle grids.

The extent of metals contamination in each spirit brand was found to show a considerable brand variation (Fig 1 and Table 3). By standard deviations alone, Cu levels had the largest variation recorded. This is followed by Zn, Al and Sr in that order. Quantitatively, Cu also had the highest concentrations recorded in any spirit brand, followed by Ba and Mn which were higher than the combined levels of Se, As, Ni, V, Cr, Sn and Sb in all brands. The lowest levels recorded were those of Pb, Co, Mo, Be, Cd, Tl and Ag. This sequence parallels the interquartile range order (IQR) given in Table 3. Note that Al and Zn trade places with Sr and Ba, respectively. Present in all the brands at more than 1 μg/L are Al, Ba, Cu, Mn and Zn (S1 Table). The brands found to contain the most metals, and were mainly Cu, were the locally distilled *Lira-Lira* spirits: AWE, BOL, NSB and TEB, and the Scottish whisky TGL (Fig 1A and Table 4). Indeed, Cu was not only disproportionally represented in these five brands (S1 Table: 97% in AWE, 92% in BOL, 100% in TEB and 95% in NSB, and 76% in TGL), but overall by 88%, mostly in TEB (55%), AWE (19%), NSB (6.3%), BOL (5.6%) and TGL (1.8%). The median level of Cu was 3.4 μg/L with the range of 0.8 to 12100 μg/L (Table 3). The mean level of Cu, which was determined to be 1079 μg/L, is greater than 2×IQR (Table 3) which implies that Cu is an outlier here. In fact, these levels of Cu exceed the WHO guideline value of 2000 μg/L [47]) and the US EPA limit for Cu intake by oral ingestion of drinking water of 1300 μg/L [48] by almost 100% in AWE (4214 ±40 μg/L) and 6000% in TEB (12100 ±1881 μg/L). Copper levels in BOL (1230 ±33 μg/L) and NSB (1397 ±985 μg/L) were close to the US EPA limit in drinking. Excluding Cu levels from the *Lira-Lira* spirits exposes the *Relax* (REX) brand as the most contaminated of all samples (Fig 1B).

**Table 3:** Summary statistics associated with 20 metals detected in 13 documented and four undocumented alcoholic beverage brands sold in northern Uganda (in μg/L). Details are in S1 Table.

	Sum	mean	SD ^a^	minimum	Q_1_ ^b^	Median	Q_3_ ^c^	maximum	IQR ^d^
Cu	19414	1079	2939	0.8	1.5	3.4	313.0	12100	311.5
Al	768.9	42.7	39.8	2.6	16.7	32.1	57.9	157.9	41.2
Sr	722.5	40.1	34.9	0.3	4.3	56.4	65.9	98.5	61.6
Zn	537.4	29.9	80.8	1.3	3.9	7.1	17.9	350.4	14.0
Ba	237.6	13.2	14.3	0.2	1.7	10.8	18.8	49.4	17.1
Mn	229.0	12.7	13.0	1.1	3.9	9.1	15.4	48.8	11.5
Se	52.2	2.9	8.3	0.1	0.2	0.8	1.9	35.8	1.7
As	22.5	1.3	2.2	0.2	0.5	0.6	1.1	9.7	0.6
Ni	20.4	1.1	1.2	0.2	0.6	0.7	1.5	5.4	0.9
V	28.7	1.0	6.7	0.0	0.0	0.0	0.0	27.6	0.0
Cr	16.2	0.9	1.7	0.1	0.2	0.3	0.4	6.4	0.3
Sn	15.9	0.9	0.9	0.1	0.4	0.5	1.0	3.2	0.7
Sb	12.9	0.7	0.4	0.3	0.5	0.6	0.9	1.6	0.4
Pb	4.1	0.2	0.3	0.1	0.1	0.1	0.2	1.1	0.1
Co	2.8	0.1	0.4	0.0	0.0	0.0	0.1	1.8	0.1
Mo	2.1	0.1	0.1	0.0	0.1	0.1	0.1	0.3	0.1
Be	0.63	0.0	0.0	0.0	0.0	0.0	0.0	0.1	0.0
Cd	0.62	0.0	0.0	0.0	0.0	0.0	0.0	0.1	0.0
Ag	0.07	0.0	0.0	0.0	0.0	0.0	0.0	0.1	0.0
Tl	0.09	0.0	0.0	0.0	0.0	0.0	0.0	0.1	0.0

^a^ SD: standard deviation

^b^ Q_1_: 25^th^ percentile.

^c^ Q_3_: 75^th^ percentile.

^d^ IQR: interquartile range (Q_3_-Q_1_).

**Table 4:** The levels of metals (μg/L) in each brand of alcohol and their associated health indices (HI). HI is listed from the most significant (top) to the least (bottom).

Sachet brand	Metal content^a^	HI metals	Ratio^b^
Ethanol	*—*	4.1 (1.0)^c^	>1 (1)^c^
TEB	12143^d^	7.3x10^-2^ ^e^	1/14
AWE	4342^d^	2.6x10^-2^ ^e^	1/38
REX	794.5	1.3x10^-2^	1/83
NSB	1479^d^	1.1x10^-2^ ^e^	1/91
BOL	1340^d^	9.6x10^-3^ ^e^	1/104
TGL	538.0^d^	5.7x10^-3^ ^e^	1/192
B5V	200.8	1.9x10^-3^	1/529
B7W	64.8	1.5x10^-3^	1/714
BEG	148.4	1.3x10^-3^	1/769
GOV	123.3	1.1x10^-3^	1/909
KPW	166.5	1.1x10^-3^	1/1000
BRG	123.3	8.9x10^-4^	1/1136
CW1	43.3	8.7x10^-4^	1/1176
ROV	164.1	8.3x10^-4^	1/1266
CW2	113.6	7.8x10^-4^	1/1300
SAG	138.9	6.9x10^-4^	1/1493
UGW	16.1	6.3x10^-4^	1/1639
V6T	148.5	5.8x10^-4^	1/1818

^a^ Sum of *n* = 20 metal levels.

^b^ Interpretation of HI values ratio: 1/*x* implies 1 person in every *x* adults may be affected); brands with the lowest ratios (hence lowest effect on adults due to metals alone) at the bottom. NB: Every individual exposed to ethanol will develop non-cancerous effect.

^c^ HI and ratio values for 40% (and 20%) (v/v) ethanol.

^d^ Copper from condensation tube (AWE, BOL, NSB and TEB) or storage container(TGL) contributes to these values as follows: TGL, 407 μg/L (76%); AWE, 4214 μg/L (97%); BOL, 1230 μg/L (92%); NSB, 1397 μg/L (94%); TEB, 12100 μg/L (100%).

^e^ Copper’s contribution to these brand’s HI values: TEB, 97%; AWE, 93%; NSB, 74%; BOL, 75%; and TGL, 45%.

Uganda Waragi (UGW) and Bond 7 Whisky (B7W), both made by EABL Group at Uganda Breweries have the lowest detectable levels of metals and, surprisingly, compares well with levels in the *Lira-Lira* spirits without Cu (AWE, BOL, NSB, and TEB; Fig 1B). UGW and B7W, two brands probably designed externally to appeal to different constituency, were also found to contain similar levels of metals, but with differing organic signatures (*unpublished* material). We believe that while different grains might have been used in the brewing step in this case, the same distillation and blending processes was employed to produce both B7W and UGW. The Chief Waragi brand sample duplicates CW1 and CW2, marketed as identical, contained interestingly differing amounts of metals (CW2: 112 μg/L and CW1: 42 μg/L, details in S1 Table). We believe a change in the manufacturing process is the likely cause of this difference since the organic signatures of CW1 and CW2 are identical (*unpublished* material).

**Fig 2:**
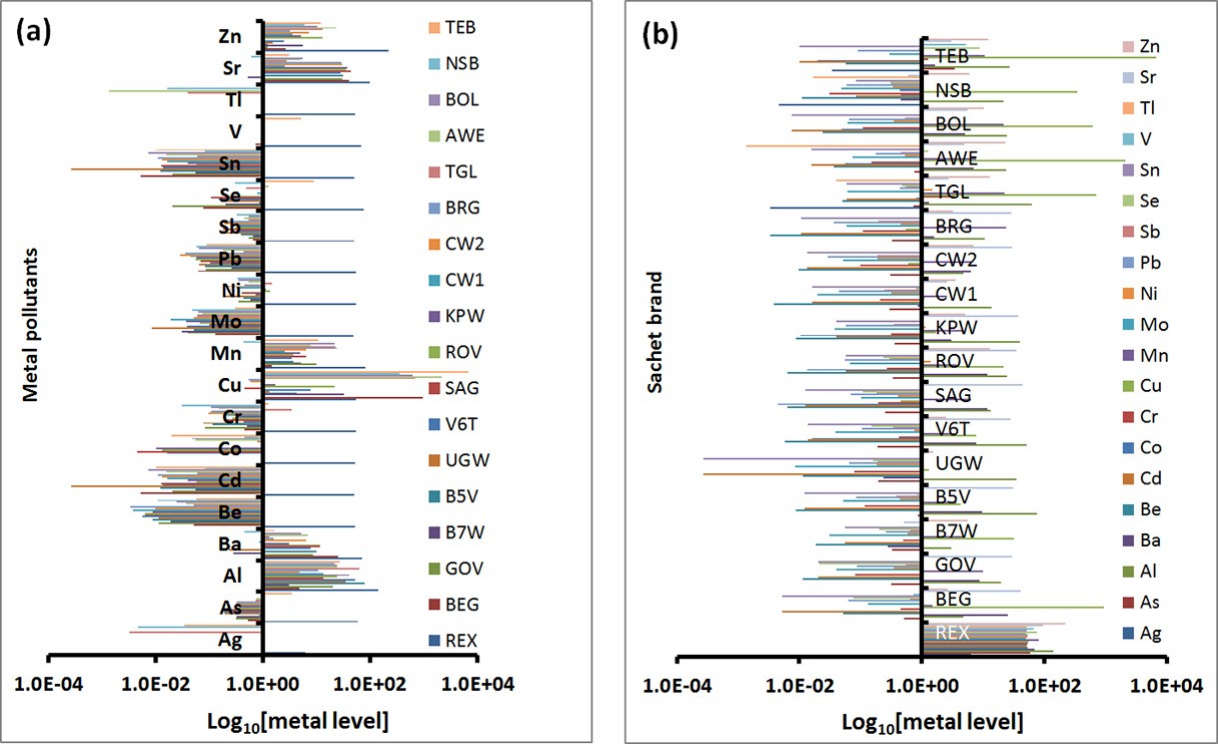
**Semi-log plots showing pictorial overview of (a) the density of contaminating metals in each sachet brand, and (b) the frequency of detecting an individual metal across the brands.** Qualitatively, REX contains the highest combination of different metals. The frequency of detecting As, Al, Ba, Be, Cd, Cr, Mn, Mo, Pb, Sb, Sn, Sr and Zn in all the brands is relatively higher than of detecting Ag, Co, Cu, Ni, Se, V and Tl.

The frequency of detecting a particular metal in each sample is shown as semi-log plots in Fig 2. The more horizontal lines are associated with a metal, the higher the chances of detecting that metal in most of the samples (Fig 2A). Similarly, the more lines a brand is associated with, the higher the probability of that brand containing the most metals (Fig 2B). It can also be seen from Fig 2 that As, Be, Cd, Co, Cr, Mo, Pb, Sb, Sn and Tl were detected mostly at low levels. Note that the blue lines representing the Relax brand (REX) in Fig 2B is linked to almost all the metals implying that this brand had the most combination of contamination. Indeed, the REX sample had 18 of the 20 metals studied (S1 Table), the highest levels being Zn (350 ± 17 μg/L), Al (158 ± 28 μg/L), Sr (99 ± 3 μg/L), Mn (49 ± 19 μg/L), Ba (42.6 ± 0.7 μg/L), Se (36 ± 17 μg/L), V (28 ± 11 μg/L), Cu (10 ± 3 μg/L) and As (10 ± 6 μg/L). The levels of Mn, Se and As were present within the WHO [51] and US EPA [52] drinking water limits (WHO/EPA: Mn, -/50 μg/L; Se, 4010/50 μg/L; As, 10/10 μg/L).

### Non-carcinogenic health risk assessment

#### *THQ* and *HI* determination

The mean levels of the 20 metals in the 18 *waragi* samples (listed in S1 Table) were used to calculate the target hazard quotients, *THQ*, from *ADI* and *RfD* as discussed in the Materials and methods section provided in S2 Table. The *THQ* values were row-percentage transformed (to accommodate for the high levels of Cu in the *Lira-Lira* spirits) then used to determine the hazard index, *HI*, for each brand (penultimate row in S2 Table) and for each metal in all brands of spirits to account for consumers with not specific brand preference (last column in S2 Table). Three lessons can be learned from the *THQ* and *HI* values as follows.

**Fig 3:**
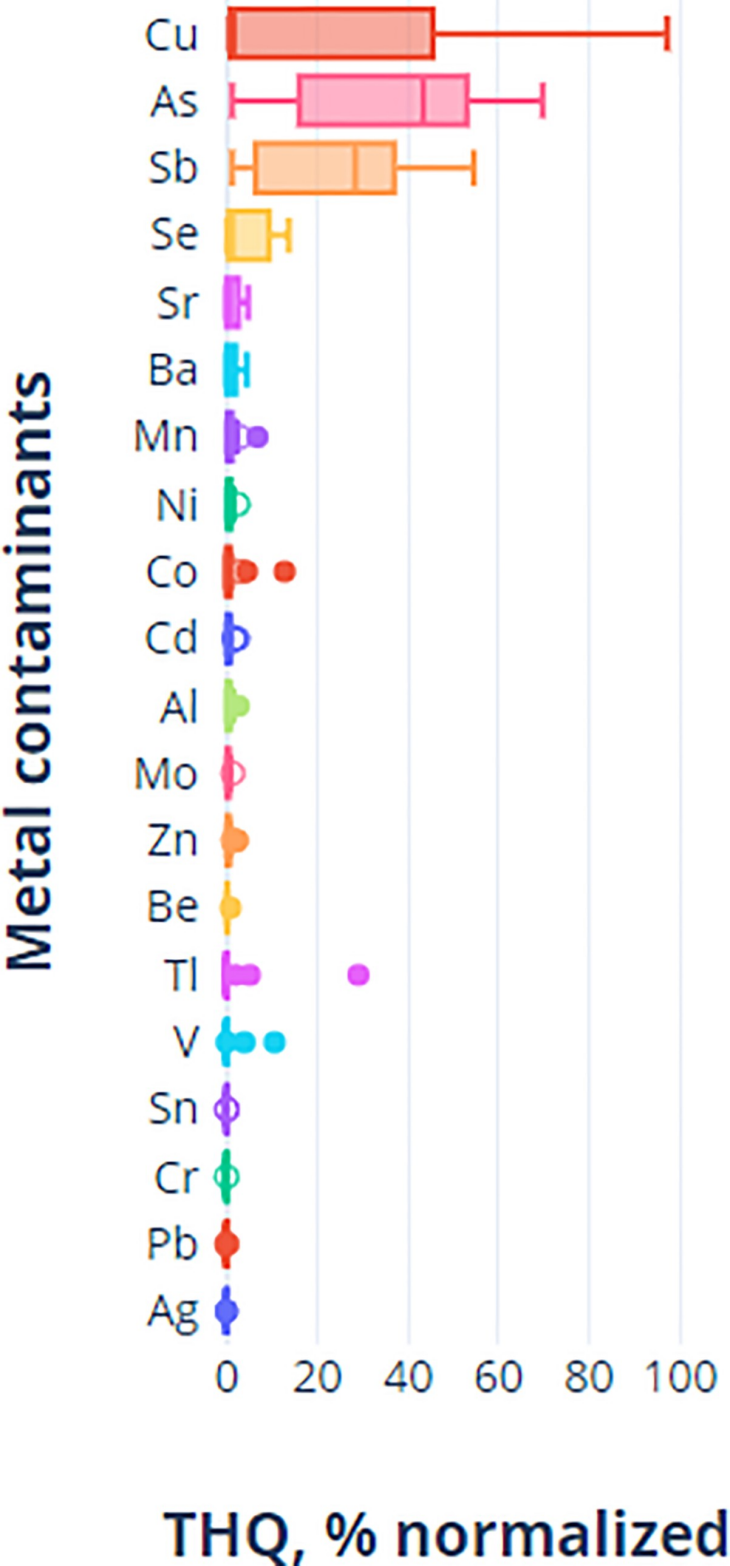
Box-Whisker plots using Plotly software for the target hazard quotient (*THQ*) data of the 20 metal ions detected in the 18 *waragi* studied. *THQ* values were row-percentage transformed to accommodate for the high levels of Cu in the *Lira-Lira* spirits). Outliers are shown as solid circles and suspected outliers as open circles. Whisker type: Standard Error; Outlier method: rounding.

First, the *THQ* results show substantial random variations in non-carcinogenic risks of consuming the 18 *waragi* spirits studied (see also Fig 3). These variations, reported in % and in the format (25^th^ -75^th^ percentile, median), are: As (19.0–51.5, 43.4), Sb (11.2–30.7, 28.4), Cu (1.3–37.4, 1.6), and Se (0.5–3.3, 1.3). Variations were also observed to a lesser extent in levels of Sr (0.0–2.6, 0.8), Mn (0.3–1.1, 0.6), Ni (0.3–0.8, 0.5), Ba (0.1–1.1, 0.4), Cd (0.2–0.7, 0.4), Al (0.1–0.6, 0.4), Zn (0.1–0.5, 0.3), Mo (0.1–0.4, 0.2), and Be (0.1–0.2, 0.1). Although detected, the *THQ* values for the remaining seven metals (Cr, Pb, V, Tl, Co, Sn and Ag) were near zero because *THQ* contribution to the toxicity of a brand by each metal is not correlated with the levels of the metals in spirits (compare values in S1 Table to values S2 Table). For ethanol itself, the *THQ* values as determined using parameters in Table 2 are 1.0 and 4.1 for the 20% and 40% (v/v) alcoholic drinks respectively. These are very high values consistent with a substance that has an *RfD* value of 62 (S2 Table; US EPA 2016 [53]); for comparison, the *RfD* values of the metals studied range from 10^−5^ to 1.5 meaning that ethanol itself poses an adverse non-carcinogenic health risk, far more than the metals detected in the spirits studied.

Secondly, the health indices (*HI* values) for ethanol, the main intoxicant in all the brands of alcoholic drinks, are about 100 to 10,000 times larger than those for metals in the 18 *waragi* samples (Table 4). This means ethanol contributes 99–100% towards the hazard index of every *waragi* studied. Ethanol should, therefore, be viewed as the single most important contributor to chronic health concern among Acoli consumers. That said, the sizes of *HI* ratios for each brand (ranked from the highest at the top to the lowest at the bottom in Table 4) offers a good measure of additional risk associated with brand preferences alone. For example, for a consumer who prefers locally distilled *Lira-Lira* spirit TEB, there is a 1 in 14 chance of being exposed to non-carcinogenic health risk (in addition to the effect of ethanol) while a consumer who prefers V6T has a 1 in 1818 of being at risk similarly.

**Fig 4:**
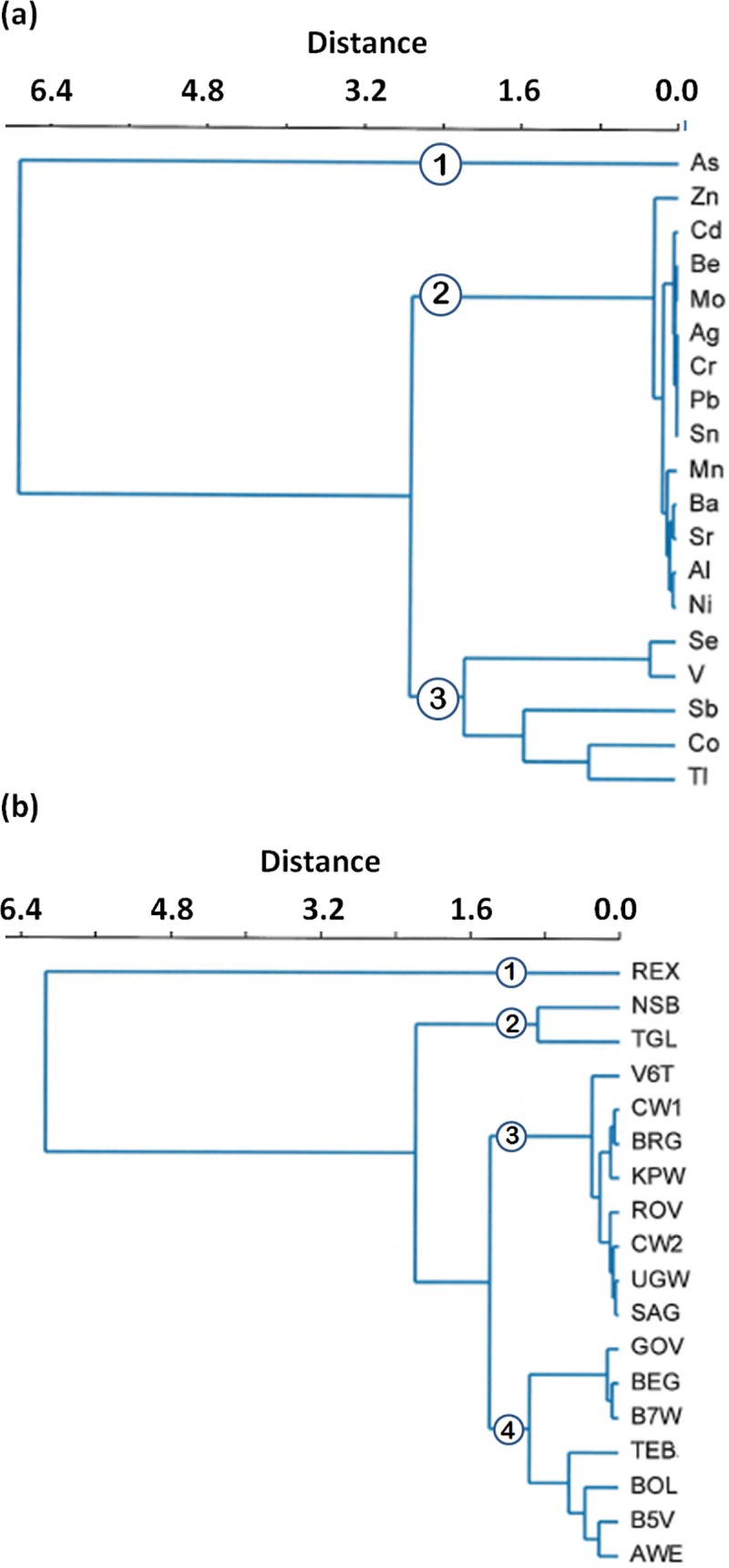
Cluster analysis of the target hazard quotient (*THQ*) data with Cu and ethanol data excluded (to provided better signal strength). (a) At an arbitrary distance of 2.4, three distinct metals clusters are observed. Cluster 1 has only one metal, As; cluster 2 contains 13 metals; cluster 3 contains Se, V, Sb, Co and Tl (Algorithm Ward’s, cophenetic correlation coefficient: 0.8952). Note that at a distance of 1.0, clusters 3 divides into three sub-clusters. (b) At an arbitrary distance of 1.0, the spirit brands cluster into five different groups (Algorithm: Ward’s, cophenetic correlation coefficient: 0.9494). REX is the only member of cluster 1; NBS and TGL are in cluster 2; cluster 3 contains eight brands: CW1, CW2, V6T, BRG, KPW, ROV, UGW, SAG; cluster 4 has GOV, BEG and B7W; and cluster 5 contains BOL, B5V and AWE.

And third, when ethanol is excluded from health risks assessment, the *Lira-Lira* spirits and the Single Malt Scotch Whisky would have the highest *HI* values, thanks to the high levels of Cu in these products. In the first place, Cu alone contributes ~76% to the *THQ* values across all brands. But when both ethanol and Cu are excluded, *Lira-Lira* spirits and the Scotch whisky would have the lowest *HI* values, comparable to UGW (the presumed ‘gold’ standard by this study). Besides Cu, other key contributions to *THQ* values of the spirit brands studied are As 12%, Sb 5%, Se and Tl, 2%, and Co and V 1%. Arsenic was mainly found in REX and contributed 62% towards the *HI* value of the Relax brand. And given that As is carcinogenic, the *Relax* brand should, therefore, be considered a serious health risk to consumers. The contributions of the remaining 13 metals (Zn, Cd, Be, Mo, Ag, Cr, Pb, Sn, Mn, Ba, Sr, Al, and Ni; Fig 4B) towards the *HI* values are negligible.

#### Determining *THQ* groupings by cluster analysis

To investigate whether natural risk groupings exist among the samples, CA was performed on a distance matrix generated from *THQ* values, the basis of which was similarities within groups and dissimilarities between different groups by Ward’s method [54]) and the squared Euclidean distance. Copper, an outlier, was excluded from this analysis. The validity of the resulting CA cluster was tested according to Clatworthy et al. [55]. Results show that natural groupings of the *THQ* values do indeed exist (see dendograms, Fig 4). In Fig 4A, all 20 metals fuse with each other to form three distinct clusters (1, 2, and 3) at a distance of 2.4. The first cluster contains only As. This means health risk associated with As is very distinct in these spirits we studied. And again, given that As was 10 times higher in the Relax brand (REX, S1 Table) than in any other brands tested, the health risk of consuming this brand of spirit is considerably distinct.

The second cluster is formed by 14 metals with negligible contributions to health concerns in the spirits tested. This cluster does not link with the third cluster containing Se, V, Sb, Co and Tl until quite a large in-between group distance of 2.8 is reached. In the third cluster, the dissimilarities within the five group members are pronounced when compared with dissimilarities within the 14-member second cluster. We interpreter this to mean each metal in the third cluster has a unique contribution to health concerns by being present in any of the spirits studied.

In Fig 4B, four distinct clusters are formed by the fusion of the 18 brands at a distance of 1.0. These risk groupings based on *THQ* values serves to show pedagogical similarities in health concerns without factoring Cu and ethanol, the two most dominant risk factors in this study. The first cluster contains only REX; the immensely large distance at which REX fused with the rest of the groups (6.2) implies that health risks presented by the *Relax* brand are unique to this brand. The second cluster contains NSB and TGL showing the distinct similarity between the southern Uganda unrecorded spirits and the Scottish whisky. The third cluster is the largest with eight brands. Note that both CW1 and CW2 group in this cluster in general, meaning the risk of consuming these two duplicates are nearly identical even though empirical data suggest otherwise. The fourth cluster contains seven members in two clearly separated sub-clusters: a closely similar risk group containing GOV, BEG and B7W, and a not so closely similar group containing TEB, BOL, B5V and AWE. The latter contains four unrecorded spirits out of five.

### Carcinogenic health risk assessment

The calculation of carcinogenic risk associated with lifetime exposure to metals in the sachet-packaged spirits collected from Acoli, Uganda was based on the ingestion of As, Pb, and Cr only, the three metals out of the 20 detected in samples whose *CSF* values are available. The carcinogenic risk was found to be 9.8 × 10^−6^, meaning one (1) individual in every 102,041 consumers of these spirits (which are laced with As, Pb, and Cr) in Acoli will develop cancer over his/her lifetime. Risks estimated on this basis are very conservative estimates, even more so given that there are no safe levels of carcinogens in biochemical terms.

## Discussion

In this study, we used a combination of analytical instrumentation and easily accessible statistical tools to show that estimating carcinogenic and non-carcinogenic health risks associated with alcohol consumption in a local sub-Saharan community with limited resources is possible. By the approach, we identified ethanol as the irrefutable risk factor in all samples collected from the Acoli region of Uganda. Ethanol is not only designated as a carcinogen by the World Health Organization in 1988 [27], but is a prime suspect by this study in causing non-carcinogenic mortality increase among consumers. That said, some examples of potential synergistic health risks associated with heavy metal poisoning are given below.

### Toxicity of heavy metals in cells

Heavy metals are known to bioaccumulate to toxic levels in soft tissues of the human body. A toxic amount of a heavy metal is defined universally as a level at which heavy metal effectively replaces some of essential metals in cells and cause stress in organs. The fact that all 20 selected heavy metals were detected in the Ugandan spirits confirms our suspicion that not only are heavy metals being consumed in the Acoli region in large amounts overtime, but that toxic bioaccumulations are taking place in the region with serious consequences. Metals such as Fe, Mn, Zn, Cr and Cu in trace amounts are central to many metabolic processes in the human body (as cofactors of enzymes essential for many biochemical transformations that influence all areas of growth and development in cells). In large quantities though, even these beneficial heavy metals can become stressors and toxins as discussed below. There are also metals such as As, Cd, and Pb which are widely distributed in the environment at toxic levels but are not known to have any beneficial values to cellular biochemical processes; As and Cd were detected in our samples.

### Biochemical roles of heavy metals in cells

The biochemical roles of heavy metals in various oxidative states in cellular destruction are well known [56–62]. Copper redox-coupled with Fe for example is implicated in enhancing oxidative stress leading to premature aging [56]. In Uganda Cu is used in the manufacture of electrical wires and in northern Uganda in particular, Cu is used in fabricating condensation tube for *Lira-Lira waragi* distillation.

To make a general case for concern in Uganda, the biochemical roles of a selection of the remaining heavy metals we studied are as follows, together with their likely sources in the country. Cadmium is implicated in the impairment of the redox equilibria of a range of biomolecules leading ultimately to cell death [57]. In Uganda, Cd is found in many household and industrial products such as storage batteries, vapor lamps, and arising from combustion. Aluminum is positively associated with the risk of cognitive impairment such as dementia and Alzheimer’s disease [58]. Aluminum in Uganda is found in containers used in the manufacture and processing of various consumables. Antimony, used for hardening Pb and in the manufacture of batteries and cables found in Uganda, is known to be absorbed and stored in the liver, kidney, and skin with Sb (III) being 10 times more toxic than Sb (V) [59]. Arsenic, likely from pesticides manufacture or distillation vessels made from poor quality Cu, is known to cause hypertension [60]. Chromium is a widely distributed metal in the earth’s crust and is industrially used in the manufacture of cars, glass, pottery and linoleum. Chromium is an essential nutrient as Cr (III) but is implicated in a wide range of in vitro and in vivo genotoxicity tests as Cr (VI). Cobalt, used in the past to stabilize froth formation in beer, causes the weakening of the heart muscles leading to as high as 50% mortality in those consuming large volumes of Co contaminated drinks [61]. Nickel, a rare metal on Earth's surface, and a toxic element that bioaccumulates in soft tissues and the bones, is used in nickel-plating, and as component of batteries and some alloys. Like Pb, the body has no use for Ni. Selenium, an essential element, is associated with several adverse health effects in humans such as hepatotoxicity, gastrointestinal disturbances, loss of nail and hair, and dermatitis [62]. Thallium, a toxic element previously used as household rodent killer, is used by electronics, glass and drugs factories. The main source of Tl is leachate from ore processing sites and waste from those factories into ground water.

All these heavy metals were detected in the sachet alcohol samples from Acoli which lends some empirical credibility to the claim that the cheaply available sachet alcohol is a major factor in the increasing mortality rate among males, the heavy consumer of alcohol in the community [23]. Of course, any factor that affects males will have dire consequences on everybody in the population directly or indirectly.

Below are some additional factors to consider in future alcohol related health assessments in this region.

### Factors driving alcohol production and consumption in Acoli

Much as it is important to establish that alcohol consumption in the Acoli population increases health risks significantly, it is equally important to determine what factors drive the high consumption of spirits in this region. Three such factors could be named here: the positive effect of increased economic activities that occurred after the war, the rise in income that came with the end of the war and the relative peace now prevailing in the region. There are also the lingering negative effects of the 20-year war [4,6–8]. For example, deprivation of livelihood in the internment camps during the war led to other downstream effects such as trauma, idleness, depression, violence, theft and others. Such negative consequences of war are believed to have carried over long after the war. Whether or not they will wear off with time and resulting in less alcohol consumption and lower health risks warrants careful observation in future. Cynically, the alcohol economy may also be driven by the dynamics of national politics in which regional marginalization is often a handy tool in the hands of those in power. For example, the increased supply of alcohol in the region may be a conscious or sub-conscious act of regional marginalization [63]. Alcohol production and consumption may also be deliberately encouraged to serve certain political interests [64]. Further studies need to be done to confirm whether these anecdotal narratives of what drives the alcohol economy in the region are true.

#### Study limitations

Several factors which are known to increase health risks of alcoholic consumption were not considered in this study. For example, the bioaccumulation rate of heavy metals in living systems, though well known, was omitted because of lack of usable data. Also lacking for inclusion are the contribution of 17 of the 20 metals studied to our cancer related health risk estimate because the US EPA is yet to establish cancer slope factors for these metals. Equally lacking are the contributions of all materials used in the production of the Ugandan spirits studied to health risks. The information of the ‘pots’ used for fermentation, distillation, or for storing the distillate before packaging, for example, is not available. Similarly, the soil in which the grains [25], cassava, or the potatoes used in the brewing process are grown, and the water used during the entire production process could account for the high level of metals and the variability observed in distillates. Water especially is prone to natural, accidental or deliberate contamination. Clouding this risk assessment from revealing the true picture of health risk in Acoli is the omission from consideration the high level of hepatitis B infections in this population (hepatitis B infections is potentially induced by ethanol [65]). The results presented here should, therefore, be taken as the minimum carcinogenic and non-carcinogenic health risks associated with alcohol consumption in Acoli, Uganda.

It is worth noting that factors such as level of education, body mass index, fasting glucose status, location of residence (urban or rural), and marital status have no statistical significance in predisposing Ugandans to the consumption of alcoholic beverages [64].

#### Conclusion and recommendations

We have presented an analytical approach for determining the health risks associated with consuming alcohol contaminated with heavy metal in this study. To our knowledge, this is the first approach of its kind to focus on addressing a root cause of alcohol-related death at the local level in a sub-Sahara African country. We find that consuming sachet packaged spirits in Uganda over a lifetime correlates with pronounced health risks in the Acoli population of Uganda. Although our finding confirms the predicted association of health risks with ingestion of heavy metals in ethanol, the primary carcinogenic and non-carcinogenic risk factor in all spirits tested is actually ethanol, the main intoxicant. This finding emphasizes the need to include ethanol as a factor in any estimates of health risks due to the consumption of alcoholic drinks in this population.

To improve the reliability of health risk assessment in Uganda in general and the Acoli region in particular, more studies need to be done. This is necessary because causal correlation between high consumption of spirits in the region and specific diseases leading to the increased mortality in Acoli population is yet to be established. More data are needed to quantify the rate of tissue and organ specific heavy metal accumulations in estimates, and to confirm the existence of a correlation between excessive alcohol consumption, heavy metal deposits and tissue/organ specific diseases in this region of Uganda. And lastly, cancer slope factors for as many orally ingested heavy metals as possible need to be determined.

Obviously, there is no silver bullet for reducing or eliminating the health risks of alcohol consumption. Nevertheless, several actions can be undertaken to ameliorate the negative effects of alcohol. First, public education about the health risks of excessive alcohol consumption must be intensified. Second, fun and productive socioeconomic activities must be developed to engage people away from excessive drinking. Idle habits acquired during the time of internment might also be playing a role here. Third, since it is unlikely that people will stop consuming alcohol despite the high health risks it poses, it is will be necessary to eliminate real or perceived marginalization to create favorable conditions against drinking as a solution to consequences of marginalization. Fourth, quality assurance in production must be improved not only to bring the quality of documented spirits in Ugandan to international standards, but to reduce precipitously the level of Cu in locally produced spirits. The latter is important because the local and traditionally produced spirits are usually cheaper than the sachets when available. Finally, new policies based on the principle that *no amount of alcohol is safe* should be adopted to address the causes of alcohol related mortality in Uganda.

## Supporting information

S1 Fig Locations of the study area in Uganda. Bolo Trading Center (BOL: 2^o^ 44' 56"N and 32^o^ 46' 45"E), Awere Trading Center (AWE: 2^o^ 41' 17"N and 32^o^ 47' 33"E), Teso Bar (TEB: 2^o^ 15' 24"N and 32^o^ 54' 07"E), a suburb of Lira Town and Nsambya (NSB, a suburb of Kampala City: 0^o^ 17' 39"N and 32^o^ 35' 20"E). TruEarth global basemap imagery reproduced with permission from TerraMetrics, Inc.
(DOCX)

S1 Table Mean metal concentrations (± standard deviation, μg/L) of duplicate measurements by ICP-MS. Values greater than 10 μg/L in bold. (a) Sachet samples. MDL: method detection limit. Brand identities and definitions are provided in Table 1. (TGL). (b) Control samples (μg/L).
(DOCX)

S2 Table Target hazard quotient (*THQ* = *ADI/RfD*) values for each metal detected in each alcohol brand used to determine the hazard index (*HI*). The percent contribution of copper (Cu) to the *HI* for AWE, BOL, NSB and TEB (the Lira-Lira drinks) shown in **bold numerals** are 93%, 75%, 74% and 97%, respectively. Note also that Cu contributes 45% to TLG’s *HI*, the reference Scotch whisky.
(DOCX)
