# Supplementary material for: Assessing the health risks of consuming ‘sachet’ alcohol in Acoli, Uganda
Source: PLoS One. 2019 Feb 27;14(2):e0212938. doi: 10.1371/journal.pone.0212938 (PMC6392317; doi:10.1371/journal.pone.0212938)
Supplement: S1 Fig — Bolo Trading Center (BOL: 2o 44' 56"N and 32o 46' 45"E), Awere Trading Center (AWE: 2o 41' 17"N and 32o 47' 33"E), Teso Bar (TEB: 2o 15' 24"N and 32o 54' 07"E), a suburb of Lira Town and Nsambya (NSB, a suburb of Kampala City: 0o 17' 39"N and 32o 35' 20"E). TruEarth global basemap imagery reproduced with permission from TerraMetrics, Inc. (DOCX) [file pone.0212938.s001.docx]

Assessing the health risks of consuming ‘sachet’ alcohol in Acoli, Uganda

Ochan Otim^1,2^*, Tom Juma^2^, Olara Otunnu^3^

^1^ Department of Humanities and Sciences, University of California - Los Angeles, Los Angeles, California, United States of America

^2^ Environmental Monitoring Division, City of Los Angeles, Playa Del Rey, California, United States of America

^3^ Former Under-Secretary-General of the United Nations, New York, New York, United States of America

**S1 Fig.** Locations of the study area in Uganda. Bolo Trading Center (BOL: 2^o^ 44' 56"N and 32^o^ 46' 45"E), Awere Trading Center (AWE: 2^o^ 41' 17"N and 32^o^ 47' 33"E), Teso Bar (TEB: 2^o^ 15' 24"N and 32^o^ 54' 07"E), a suburb of Lira Town and Nsambya (NSB, a suburb of Kampala City: 0^o^ 17' 39"N and 32^o^ 35' 20"E). TruEarth global basemap imagery reproduced with permission from TerraMetrics, Inc.


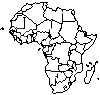

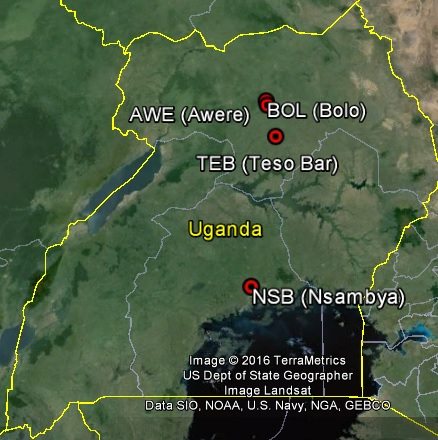


AFRICA
