# Supplementary material for: Assessing the health risks of consuming ‘sachet’ alcohol in Acoli, Uganda
Source: PLoS One. 2019 Feb 27;14(2):e0212938. doi: 10.1371/journal.pone.0212938 (PMC6392317; doi:10.1371/journal.pone.0212938)
Supplement: S1 Table — Values greater than 10 μg/L in bold. (a) Sachet samples. MDL: method detection limit. Brand identities and definitions are provided in Table 1. (TGL). (b) Control samples (μg/L). (DOCX) [file pone.0212938.s002.docx]

**Assessing the health risks of consuming ‘sachet’ alcohol in Acoli, Uganda**

*Page 1 of 2*

Ochan Otim^1,2^*, Tom Juma^2^ and Olara Otunnu^3^

^1^ *University of California, Los Angeles, Department of Humanities and Sciences, 10995 Le Conte Ave., Los Angeles, CA 90024.*

^2^ *City of Los Angeles, Environmental Monitoring Division, 12000 Vista Del Mar, Playa Del Rey, CA. 90293.*

^3^ *Former Under-Secretary-General of the United Nations, 405 East 42nd Street, New York, NY, 10017, USA.*

* Corresponding author. Tel.: +1 310 648 5835; fax: +1 310 648 5731. *E-mail address:* ootim@g.ucla.edu. ORCID: https://orcid.org/0000-0001-7272-4356

**S1 Table**. Mean metal concentrations (± standard deviation, μg/L) of duplicate measurements by ICP-MS. Values greater than 10 μg/L in bold.

**(a)** Sachet samples. MDL: method detection limit. Brand identities and definitions are provided in Table 1.

**MDL REX BEG GOV B7W B5V UGW V6T SAG ROV KPW CW1 CW2 BRG**

**^107^Ag 4E-3** 0.01±0.00 -0.02±0.00 -0.03±0.00 -0.03±0.00 -0.03±0.00 -0.03±0.00 -0.01±0.00 -0.01±0.00 -0.01±0.00 -0.01±0.00 -0.01±0.00 -0.01±0.00 -0.01±0.00

**^75^As 0.21** 9.74±5.95 0.56±0.07 0.63±0.09 0.58±0.05 1.68±0.01 0.47±0.13 0.20±0.02 0.45±0.06 0.44±0.01 0.68±0.04 0.59±0.05 0.50±0.10 0.60±0.06

**^27^Al 0.61 157.85±28.1** 2.61±0.24 **33.27±9.11** **13.13±9.99 97.86±65.54 6.67±1.64 62.94±9.07 16.89±1.46 38.08±7.25 69.88±5.57 20.54±1.66 10.51±3.70 16.69±1.49**

**^137^Ba 0.02 42.62±0.74** **49.36±0.88 15.74±1.31** 0.50±0.06 **19.79±0.28** 0.22±0.04 **15.89±0.82 21.99±0.71 22.42±1.39** 4.76±0.13 1.58±0.01 **12.86±0.27** 2.87±0.01

**^9^Be 0.03** 0.13±0.07 0.10±0.00 0.02±0.01 0.03±0.00 0.01±0.00 0.02±0.00 0.01±0.00 0.01±0.00 0.01±0.01 0.01±0.00 0.01±0.01 0.01±0.00 0.01±0.01

**^111^Cd 0.02** 0.03±0.00 0.01±0.01 0.03±0.00 0.10±0.01 0.02±0.00 0.07±0.10 0.02±0.01 0.02±0.00 0.06±0.06 0.02±0.00 0.03±0.00 0.02±0.01 0.01±0.00

**^59^Co 0.01** 0.48±0.02 -0.03±0.00 -0.03±0.00 -0.04±0.00 -0.03±0.01 -0.06±0.00 0.00±0.00 0.01±0.00 0.01±0.03 -0.01±0.00 -0.03±0.00 -0.03±0.00 -0.01±0.01

**^52^Cr 0.07** 4.74±2.38 0.63±0.02 0.13±0.03 1.01±0.04 0.18±0.03 0.18±0.06 0.32±0.11 0.26±0.03 0.42±0.09 0.45±0.14 0.21±0.09 0.15±0.06 0.17±0.02

**^63^Cu 0.03 10.22±2.51** 3.82±0.59 1.45±0.02 **31.48±0.28** 7.48±1.12 1.60±0.14 1.17±0.07 0.82±0.02 2.23±0.33 2.89±0.25 1.96±0.03 1.22±0.07 1.05±0.03

**^55^Mn 0.14 48.76±18.8** 1.08±1.04 **11.73±9.87** 1.24±0.12 6.14±1.64 1.12±0.87 3.70±2.23 **12.34±2.46** 4.78±2.42 7.34±0.29 3.58±1.79 **10.87±0.25 35.70±17.42**

**^98^Mo 0.03** 0.29±0.22 0.14±0.02 0.08±0.03 0.06±0.00 0.10±0.01 0.03±0.03 0.06±0.01 0.19±0.00 0.12±0.01 0.06±0.00 0.04±0.01 0.10±0.00 0.12±0.00

**^60^Ni 0.14** 5.44±0.14 1.67±0.01 0.54±0.21 1.15±0.18 0.52±0.25 0.20±0.12 0.59±0.23 0.82±0.32 1.68±1.44 0.66±0.29 0.32±0.05 0.51±0.26 0.62±0.04

**^207^Pb 0.01** 1.06±0.08 0.12±0.01 0.13±0.05 0.52±0.03 0.09±0.07 0.07±0.01 0.17±0.01 0.11±0.02 0.09±0.01 0.10±0.00 0.08±0.00 0.07±0.02 0.05±0.00

**^123^Sb 0.01** 0.64±0.20 1.07±0.21 0.89±0.00 1.19±0.15 0.61±0.03 0.36±0.01 0.52±0.02 0.33±0.02 0.50±0.13 0.53±0.05 0.45±0.01 0.37±0.00 0.35±0.05

**^78^Se 0.43 35.79±17.0** 0.18±0.04 0.06±0.08 0.14±0.01 1.54±**0.18** 0.06±0.11 0.18±0.09 0.25±0.17 0.28±0.15 2.09±0.19 1.75±0.47 1.54±0.03 1.94±0.31

**^118^Sn 0.02** 0.03±0.00 0.01±0.01 0.03±0.00 0.10±0.01 0.02±0.00 0.07±0.10 0.02±0.01 0.02±0.00 0.06±0.06 0.02±0.00 0.03±0.00 0.02±0.01 0.01±0.00

**^51^V 0.28 27.59±10.71** 1.07±0.02 -0.65±0.19 -1.65±0.01 -0.68±0.02 -0.83±0.08 -1.05±0.26 -0.80±0.33 -0.90±0.19 -0.81±0.14 -0.91±0.03 -0.86±0.01 -0.67±0.08

**^205^Tl 0.01** 0.01±0.01 -0.02±0.00 -0.02±0.00 -0.02±0.00 -0.02±0.00 -0.02±0.00 -0.01±0.00 0.00±0.00 -0.01±0.00 -0.01±0.00 -0.01±0.00 -0.01±0.00 -0.01±0.01

**^86^Sr ⎯ 98.45±2.62** **80.57±2.35 55.71±2.44** 0.61±0.03 **62.96±1.97** 0.27±0.02 **58.44±3.16 82.06±2.68 66.91±4.59 69.15±0.52** 4.86±0.28 **60.38±0.80 57.00±0.09**

**^66^Zn 0.32 350.35±16.90** 5.00±0.40 1.82±0.31 9.81±0.18 1.26±1.47 2.68±1.47 3.51±0.43 1.92±0.50 24.19±2.18 7.46±0.83 6.53±0.25 14.10±0.31 5.94±0.33

**Sum** 794.6 148.3 122.6 63.0 200.0 15.2 147.4 138.1 163.1 165.6 42.4 112.7 122.6

**Mean** 39.71 7.40 6.08 3.00 9.97 0.66 7.33 6.88 8.07 8.26 2.08 5.62 6.12

**SD** 81.33 19.86 13.90 7.38 24.54 1.55 18.14 18.32 16.95 20.55 4.61 13.44 14.32

*Page 2 of 2*

**(b)** Control samples (μg/L)**.**

**TGL**^a^ **AWE**^b^ **BOL**^b^ **NSB**^b,c^ **TEB**^b^ **WHO guidelines US EPA limits**^e^

**^107^Ag** 0.00±0.00 -0.01±0.00 -0.03±0.00 0.00±0.01 0.06±0.00 ⎯ 100

**^75^As** 0.67±0.17 1.27±0.24 1.98±0.04 0.31±0.68 1.22±0.53 10 10

**^27^Al 87.72±35.19 42.86±5.65 40.02±0.06 30.91±14.69 20.51±7.14** 900 200

**^137^Ba** 1.41±0.10 **13.19±0.12** 8.81±0.10 1.69±1.18 1.92±0.83 700 2000

**^9^Be** 0.07±0.01 0.06±0.01 0.03±0.01 0.04±0.03 0.05±0.01 12 4

**^111^Cd** 0.08±0.01 0.02±0.01 0.01±0.00 0.05±0.03 0.01±0.00 3 5

**^59^Co** 0.30±0.03 0.11±0.00 0.09±0.00 1.80±1.27 0.01±0.01 ⎯ ⎯

**^52^Cr** 6.39±0.47 0.24±0.05 0.20±0.02 0.13±0.18 0.37±0.27 50 100

**^63^Cu 406.9±14.5**  **4214±40**  **1230±33**  **1397±985**  **12100±2** 2000 1300

**^55^Mn 16.06±1.51 13.38±0.81 27.22±9.07 19.38±27.41** 4.63±2.64 400 50

**^98^Mo** 0.10±0.02 0.14±0.01 0.09±0.01 0.08±0.01 0.30±0.11 70 ⎯

**^60^Ni** 1.73±0.74 0.72±0.05 0.59±0.15 1.07±0.72 1.61±0.18 70 ⎯

**^207^Pb** 0.72±0.04 0.32±0.02 0.09±0.02 0.18±0.11 0.11±0.01 10 15

**^123^Sb** 0.77±0.14 0.88±0.00 1.08±0.01 0.78±0.21 1.62±0.09 20 6

**^78^Se** 0.22±0.05 2.43±0.09 2.07±0.26 1.06±0.79 0.60±0.00 40 50

**^118^Sn** 0.08±0.01 0.02±0.01 0.01±0.00 0.05±0.03 0.01±0.00 ⎯ ⎯

**^51^V** -0.37±0.01 -0.24±0.25 -0.32±0.02 -0.54±0.12 0.07±0.42 ⎯ ⎯

**^205^Tl** 0.07±0.00 0.00±0.00 -0.01±0.00 0.02±0.01 -0.01±0.00 ⎯ 2

**^86^Sr** 4.13±0.10 7.01±0.17 8.51±0.42 2.16±1.39 3.38±0.14 ⎯ ⎯

**^66^Zn** 8.84±1.11 **45.93±0.26 19.20±1.36 22.02±16.18** 6.80±0.66 ⎯ ⎯

**Sum** 537.6 4342.0 1339.3 1478.9 12143.4

**Mean** 26.79 217.09 66.96 73.90 607.16

**SD** 89.24 916.93 266.92 303.62 2636.64

Key

^a^ TGL: Control, used to compare against western packaged gin.

^b^AWE, BOL, NSB, TEB: Controls; locally distilled and distributed alcohol beverages for comparison.

^c^ NSB, a regional comparative control.

^d^ World Health Organization. 2011. Guidelines for Drinking-water Quality. 4th Ed. [47]

^e^United States Environmental Protection Agency. 2009. National Primary Drinking Water Regulation [48].
