# Supplementary material for: Assessing the health risks of consuming ‘sachet’ alcohol in Acoli, Uganda
Source: PLoS One. 2019 Feb 27;14(2):e0212938. doi: 10.1371/journal.pone.0212938 (PMC6392317; doi:10.1371/journal.pone.0212938)
Supplement: S2 Table — The percent contribution of copper (Cu) to the HI for AWE, BOL, NSB and TEB (the Lira-Lira drinks) shown in bold numerals are 93%, 75%, 74% and 97%, respectively. Note also that Cu contributes 45% to TLG’s HI, the reference Scotch whisky. (DOCX) [file pone.0212938.s003.docx]

**Assessing the health risks of consuming ‘sachet’ alcohol in Acoli, Uganda**

Ochan Otim^1,2^*, Tom Juma^2^ and Olara Otunnu^3^

^1^ *University of California, Los Angeles, Department of Humanities and Sciences, 10995 Le Conte Ave., Los Angeles, CA 90024.*

^2^ *City of Los Angeles, Environmental Monitoring Division, 12000 Vista Del Mar, Playa Del Rey, CA. 90293.*

^3^ *Former Under-Secretary-General of the United Nations, 405 East 42nd Street, New York, NY, 10017, USA.*

* Corresponding author. Tel.: +1 310 648 5835; fax: +1 310 648 5731. *E-mail address:* ootim@g.ucla.edu. ORCID: https://orcid.org/0000-0001-7272-4356

**S2 Table.** Target hazard quotient (THQ = ADI/RfD) values for each metal detected in each alcohol brand used to determine the hazard index (HI). The percent contribution of copper (Cu) to the HI for AWE, BOL, NSB and TEB (the *Lira-Lira* drinks) shown in **bold** numerals is 93%, 75%, 74% and 97%, respectively. Note also that Cu contributes 45% to TLG’s HI, the reference Scotch whisky.

**Alcoholic beverage samples**

**Metals RfD**^a^ **REX BEG GOV B7W B5V UGW TGL**^b^ **V6T SAG ROV KPW CW1 CW2 BRG AWE**^b^ **BOL**^b^ **NSB**^b^ **TEB**^b^ **HI**^c^

**^107^Ag** **5.0E-03** 3.5E-07 0.0E+00 0.0E+00 0.0E+00 0.0E+00 0.0E+00 0.0E+00 0.0E+00 0.0E+00 0.0E+00 0.0E+00 0.0E+00 0.0E+00 0.0E+00 0.0E+00 0.0E+00 1.9E-07 2.9E-06 **3.4E-06**

**^75^As** **3.0E-04** 7.6E-03 4.4E-04 4.9E-04 4.5E-04 1.3E-03 3.6E-04 5.2E-04 1.5E-04 3.5E-04 3.4E-04 5.3E-04 4.6E-04 3.9E-04 4.6E-04 9.9E-04 1.5E-03 2.4E-04 9.5E-04 **1.8E-02**

**^27^Al** **1.0** 3.7E-05 6.1E-07 7.8E-06 3.1E-06 2.3E-05 1.6E-06 2.1E-05 1.5E-05 3.9E-06 8.9E-06 1.6E-05 4.8E-06 2.5E-06 3.9E-06 1.0E-05 9.4E-06 7.2E-06 4.8E-06 **1.8E-04**

**^137^Ba** **0.2** 5.0E-05 5.8E-05 1.8E-05 5.9E-07 2.3E-05 2.6E-07 1.7E-06 1.9E-05 2.6E-05 2.6E-05 5.6E-06 1.9E-06 1.5E-05 3.4E-06 1.5E-05 1.0E-05 2.0E-06 2.2E-06 **2.8E-04**

^9^**Be** **2.0E-03** 1.5E-05 1.1E-05 2.7E-06 3.2E-06 1.4E-06 2.0E-06 8.2E-06 7.6E-07 1.2E-06 8.2E-07 1.5E-06 1.3E-06 1.7E-06 1.4E-06 7.4E-06 3.7E-06 4.4E-06 5.7E-06 **7.4E-05**

^111^**Cd** **1.0E-03** 7.5E-06 3.3E-06 7.7E-06 2.4E-05 3.9E-06 1.5E-05 1.8E-05 5.7E-06 3.9E-06 1.5E-05 4.6E-06 6.0E-06 3.9E-06 2.6E-06 5.5E-06 3.4E-06 1.1E-05 3.2E-06 **1.4E-04**

^59^**Co** **3.0E-04** 3.8E-04 0.0E+00 0.0E+00 0.0E+00 0.0E+00 0.0E+00 2.3E-04 3.9E-07 7.8E-06 6.2E-06 0.0E+00 0.0E+00 0.0E+00 0.0E+00 8.3E-05 7.1E-05 1.4E-03 9.4E-06 **2.2E-03**

^52^**Cr** **1.5** 7.4E-07 9.7E-08 2.0E-08 1.6E-07 2.8E-08 2.7E-08 1.0E-06 5.0E-08 4.1E-08 6.6E-08 7.0E-08 3.3E-08 2.3E-08 2.7E-08 3.7E-08 3.2E-08 2.0E-08 5.8E-08 **2.5E-06**

^63^**Cu** **0.04** 6.0E-05 2.2E-05 8.5E-06 1.8E-04 4.4E-05 9.3E-06 **2.4E-03** 6.9E-06 4.8E-06 1.3E-05 1.7E-05 1.1E-05 7.1E-06 6.1E-06 **2.5E-02 7.2E-03 8.2E-03 7.1E-02** **1.1E-01**

^55^**Mn** **0.14** 8.1E-05 1.8E-06 2.0E-05 2.1E-06 1.0E-05 1.9E-06 2.7E-05 6.2E-06 2.1E-05 8.0E-06 1.2E-05 6.0E-06 1.8E-05 6.0E-05 2.2E-05 4.5E-05 3.2E-05 7.7E-06 **3.8E-04**

**^98^Mo** **5.0E-03** 1.4E-05 6.7E-06 3.8E-06 2.7E-06 4.5E-06 1.5E-06 4.4E-06 2.6E-06 9.1E-06 5.4E-06 3.0E-06 1.6E-06 4.6E-06 5.4E-06 6.4E-06 4.1E-06 3.6E-06 1.4E-05 **9.7E-05**

**^60^Ni** **0.02** 6.4E-05 1.9E-05 6.3E-06 1.3E-05 6.1E-06 2.3E-06 2.0E-05 6.9E-06 9.6E-06 2.0E-05 7.7E-06 3.7E-06 6.0E-06 7.3E-06 8.4E-06 6.9E-06 1.3E-05 1.9E-05 **2.4E-04**

**^207^Pb** **1.5** 1.6E-07 1.8E-08 2.1E-08 8.1E-08 1.5E-08 1.1E-08 1.1E-07 2.6E-08 1.8E-08 1.3E-08 1.6E-08 1.3E-08 1.1E-08 8.1E-09 5.1E-08 1.4E-08 2.8E-08 1.8E-08 **6.4E-07**

**^123^Sb** **4.0E-04** 3.7E-04 6.2E-04 5.2E-04 7.0E-04 3.5E-04 2.1E-04 4.5E-04 3.0E-04 1.9E-04 2.9E-04 3.1E-04 2.6E-04 2.2E-04 2.0E-04 5.1E-04 6.3E-04 4.6E-04 9.5E-04 **7.6E-03**

**^78^Se** **5.0E-03** 1.7E-03 8.4E-06 2.9E-06 6.5E-06 7.2E-05 2.9E-06 1.0E-05 8.6E-06 1.2E-05 1.3E-05 9.8E-05 8.2E-05 7.2E-05 9.1E-05 1.1E-04 9.7E-05 4.9E-05 2.8E-05 **2.4E-03**

**^118^Sn** **0.6** 1.5E-07 1.5E-07 4.2E-07 1.3E-06 2.2E-07 8.2E-07 7.4E-07 3.0E-07 1.8E-07 7.3E-07 1.4E-07 3.1E-07 1.7E-07 6.8E-08 6.9E-08 4.9E-08 3.7E-07 4.4E-08 **6.2E-06**

**^51^V** **5.0E-03** 1.3E-03 5.0E-05 0.0E+00 0.0E+00 0.0E+00 0.0E+00 0.0E+00 0.0E+00 0.0E+00 0.0E+00 0.0E+00 0.0E+00 0.0E+00 0.0E+00 0.0E+00 0.0E+00 0.0E+00 3.1E-06 **1.3E-03**

**^205^Tl** **1.0E-05** 2.3E-04 0.0E+00 0.0E+00 0.0E+00 0.0E+00 0.0E+00 1.5E-03 0.0E+00 0.0E+00 0.0E+00 0.0E+00 0.0E+00 0.0E+00 0.0E+00 0.0E+00 0.0E+00 5.6E-04 0.0E+00 **2.3E-03**

**^86^Sr** **0.6** 3.8E-05 3.1E-05 2.2E-05 2.4E-07 2.5E-05 1.1E-07 1.6E-06 2.3E-05 3.2E-05 2.6E-05 2.7E-05 1.9E-06 2.4E-05 2.2E-05 2.7E-06 3.3E-06 8.4E-07 1.3E-06 **2.8E-04**

**^66^Zn** **0.3** 2.7E-04 3.9E-06 1.4E-06 7.6E-06 9.8E-07 2.1E-06 6.9E-06 2.7E-06 1.5E-06 1.9E-05 5.8E-06 5.1E-06 1.1E-05 4.6E-06 3.6E-05 1.5E-05 1.7E-05 5.3E-06 **4.2E-04**

***Ethanol*** ***62.0*** *1.0 4.1 4.1 4.1 4.1 4.1 4.1 4.1 4.1 4.1 4.1 4.1 4.1 4.1 4.1 4.1 4.1 4.1* ***71***

**HI**^c^ 1.2E-02 1.3E-03 1.1E-03 1.4E-03 1.9E-03 6.1E-04 5.2E-03 5.5E-04 6.7E-04 7.9E-04 1.0E-03 8.5E-04 7.7E-04 8.8E-04 2.6E-02 9.6E-03 1.1E-02 7.3E-02 **1.5E-01**

**HI/EtOH^d^** **1.0 4.1 4.1 4.1 4.1 4.1 4.1 4.1 4.1 4.1 4.1 4.1 4.1 4.1 4.1 4.1 4.1 4.1 71**

^a^ **RfD:** Oral reference dose of individual metal (US EPA 2016). ^b^ Reference drinks, not packaged in sachet. ^c^ **HI:** Hazard Index (ƩTHQ) per brand in second row from bottom, and per metal in right most column. THQs for AWE, BOL, NSB and TEB are based on 20% (v/v) alcohol. ^d^ **HI/EtOH**: ethanol HI values included. HI for REX is low because REX is 20% alcohol (*vs* 40% in other brands).
